# Diagnostic Value of sIL-2R, TNF-α and PCT for Sepsis Infection in Patients With Closed Abdominal Injury Complicated With Severe Multiple Abdominal Injuries

**DOI:** 10.3389/fimmu.2021.741268

**Published:** 2021-10-22

**Authors:** Guang-hua Zhai, Wei Zhang, Ze Xiang, Li-Zhen He, Wei-wei Wang, Jian Wu, An-quan Shang

**Affiliations:** ^1^ Department of Clinical Laboratory, The Affiliated Suzhou Hospital of Nanjing Medical University, Suzhou Municipal Hospital, Gusu School, Nanjing Medical University, Suzhou, China; ^2^ Department of Laboratory Medicine, Jiaozuo Fifth People’s Hospital, Jiaozuo, China; ^3^ Zhejiang University School of Medicine, Hangzhou, China; ^4^ Department of Laboratory, Jiaozuo Second People’s Hospital, Jiaozuo, China; ^5^ Department of Laboratory Medicine, The First Affiliated Hospital of Henan Polytechnic University, Henan, China; ^6^ Department of Pathology, Tinghu People’s Hospital of Yancheng City, Yancheng, China; ^7^ Department of Laboratory Medicine, Shanghai Tongji Hospital, School of Medicine, Tongji University, Shanghai, China

**Keywords:** sIL-2R, TNF-α, PCT, sepsis, closed abdominal injury complicated with severe multiple abdominal injuries, diagnostic value

## Abstract

**Objective:**

We aimed to evaluate the diagnostic value of soluble interleukin-2 receptor (sIL-2R), tumor necrosis factor-α (TNF-α), procalcitonin (PCT), and combined detection for sepsis infection in patients with closed abdominal injury complicated with severe multiple abdominal injuries.

**Patients and Methods:**

One hundred forty patients with closed abdominal injury complicated with severe multiple abdominal injuries who were diagnosed and treated from 2015 to 2020 were divided into a sepsis group (*n* = 70) and an infection group (*n* = 70).

**Results:**

The levels of sIL-2R, TNF-α, and PCT in the sepsis group were higher than those in the infection group (*p* < 0.05). The receiver operating characteristic (ROC) curve showed that the areas under the ROC curve (AUCs) of sIL-2R, TNF-α, PCT and sIL-2R+TNF-a+PCT were 0.827, 0.781, 0.821, and 0.846, respectively, which were higher than those of white blood cells (WBC, 0.712), C-reactive protein (CRP, 0.766), serum amyloid A (SAA, 0.666), and IL-6 (0.735). The AUC of the three combined tests was higher than that of TNF-α, and the difference was statistically significant (*p* < 0.05). There was no significant difference in the AUCs of sIL-2R and TNF-α, sIL-2R and PCT, TNF-α and PCT, the three combined tests and sIL-2R, and the three combined tests and PCT (*p* > 0.05). When the median was used as the cut point, the corrected sIL-2R, TNF-α, and PCT of the high-level group were not better than those of the low-level group (*p* > 0.05). When the four groups were classified by using quantile as the cut point, the OR risk values of high levels of TNF-α and PCT (Q4) and the low level of PCT (Q1) after correction were 7.991 and 21.76, respectively, with statistical significance (*p* < 0.05).

**Conclusions:**

The detection of sIL-2R, TNF-α, and PCT has good value in the diagnosis of sepsis infection in patients with closed abdominal injury complicated with severe multiple abdominal injuries. The high concentrations of PCT and TNF-α can be used as predictors of the risk of septic infection.

## Introduction

Closed abdominal injury with severe multiple traumas is characterized by complex and hidden conditions, and most patients are accompanied by injuries to the brain, chest, and limbs (1). The prognosis of closed abdominal injury depends on the presence or absence of visceral injury, which is characterized by persistent vomiting, nausea, abdominal pain, internal bleeding, and peritoneal irritation in terms of clinical symptoms (2). For patients with closed abdominal injuries accompanied by severe multiple injuries, besides abdominal injuries, fractures, brain injuries, etc., are also present (3). The accompanying multiple injuries may conceal the actual physical signs of patients, thus increasing the difficulty of clinical diagnosis. In the case of closed abdominal injury combined with multiple systemic injuries, the patients’ injuries are severe and complex, including shock, sepsis, coma, dyspnea, and other symptoms in the early stage, which makes the diagnosis and treatment more difficult and leads to higher mortality (4).

Sepsis is a systemic inflammatory response syndrome caused by severe infection. The main pathogens of sepsis are bacteria, followed by fungi, viruses, and parasites (5). Sepsis is a major cause of death worldwide, with a high incidence, which can cause damage to important organs such as the heart, lung, and kidney. Sepsis has been reported to cause about 25%–50% of deaths in the United States, Europe, and South America (6). Blood culture is considered as the “golden criteria” for diagnosing septic infection (7), but it has been reported (8) that only half of patients suspected of sepsis were found to be infected by pathogenic bacteria; there were also problems such as contamination of normal skin bacteria and long culture times, leading to the inability to obtain results in time (9). Therefore, the early diagnosis of sepsis and targeted treatment within a few hours of the first diagnosis are extremely important (10). Sepsis is a potentially fatal disease defined as an infection-related syndrome exacerbated by acute organ failure. Organ dysfunction is caused by an acute change in the Sequential Organ Failure Assessment (SOFA) score of ≥2 points, i.e., sepsis = infection + SOFA score ≥2 points (11). Although the diagnostic criteria for sepsis have been established, the early diagnosis of sepsis is still difficult due to the unclear primary infectious focus and the vague definition of sepsis syndrome (12). Several scholars believe that there is a lag in the SOFA score, and most patients are in the late stage of sepsis when they are diagnosed using the SOFA score (13). Therefore, a simple diagnostic index with high diagnostic performance in the early stage of sepsis remains to be studied.

Soluble interleukin 2 receptor (sIL-2R) is a nonspecific indicator produced by lymphocytes under conditions such as malignant tumors and infection, which can reflect diseases related to lymphocyte activation and is also a marker of the activation of the immune system (14). Tumor necrosis factor-α (TNF-α) is a major pro-inflammatory cytokine that plays a key role in antimicrobial and anti-inflammatory responses through mechanisms such as the activation of white blood cells, cell proliferation, differentiation, and apoptosis of lymphocytes (15, 16). A large number of cytokines are produced in the process of infection, among which TNF-a plays a powerful immune regulatory role in host immune response (17). Serum procalcitonin (PCT) is a type of calcitonin propeptide that is elevated during inflammation and infection, and it is also considered as a diagnostic marker for early infection (18). One of the recognized mechanisms of sepsis is that its occurrence and the development process are caused by an imbalance in the anti-inflammatory response. Sepsis can promote the secretion of inflammatory factors in the early stage. At present, there are no reports on the diagnostic value of sIL-2R, TNF-α, and PCT in sepsis patients with closed abdominal injury. Based on the Third International Consensus Definitions for Sepsis and Septic Shock diagnostic criteria (11), this study intended to evaluate the diagnostic value of sIL-2R, TNF-α, PCT, and their combined detection in sepsis patients with closed abdominal injury complicated with severe multiple abdominal injuries. The optimal threshold value of infection in patients with sepsis was determined in this study, and the influence of sIL-2R, TNF-α, and PCT was evaluated, which also provides reference for the early diagnosis of sepsis in patients with closed abdominal injury complicated with severe multiple abdominal injuries.

## Materials and Methods

### Patients and Clinical Information

We retrospectively analyzed 140 patients with closed abdominal injury complicated with severe multiple abdominal injuries treated in the Fifth People’s Hospital of Jiaozuo City, The First Affiliated Hospital of Henan University of Technology, the First People’s Hospital of Yancheng City, and the Tinghu People’s Hospital of Yancheng City from 2015 to 2020, including 70 patients with sepsis (sepsis group from intensive care units) and 70 patients without sepsis but with local inflammatory infection (infection group from general surgery). All the medical records were confirmed by clinical symptoms, B-ultrasound, X-ray films, and laboratory, among others. The locations of abdominal injuries were the pancreas in 13 cases, the duodenum in 15 cases, colon in 32 cases, liver in 13 cases, small intestine in 19 cases, and the spleen in 48 cases; 98 cases had multiple organ injuries and 42 cases had single organ injuries. The diagnosis of sepsis was based on the Third International Consensus Diagnostic Criteria for Sepsis and Septic Shock 3.0, published in 2016.

The infection group showed two or more of the following signs: body temperature, >38°C or <36°C; heart rate, >90 bpm; respiratory rate of >20 times/min or arterial blood carbon dioxide partial pressure of <32 mmHg; and peripheral blood leukocytes, >12 × 10^9^/L or <4 × 10^9^/L. The criteria for the sepsis group were in accordance with the signs in the infection group and a SOFA score ≥2.

The exclusion criteria were as follows: patients with solid cancer, hematologic disease, organ transplantation, with missing clinical and laboratory data, treated with antibiotics, and patients with immune deficiency.

The baseline clinical data of 140 enrolled patients were collected from medical records, which included age, gender, sIL-2R, TNF-α, PCT, white blood cells (WBC), C-reactive protein (CRP), and serum amyloid A (SAA), IL-6, as well as Acute Physiology and Chronic Health Assessment (APACHE II). The included detection indexes were based on the serum samples collected for the first time within 24 h of a patient’s visit. SAA was detected by kinetics nephelometry. sIL-2R, TNF-α, and IL-6 were detected with ELISA. PCT was detected using immunofluorescence chromatography. All specimens were enrolled after obtaining informed consent from the patients or their family. Written informed consent from the participants was obtained. The study was approved by the ethics committees of the Fifth People’s Hospital of Jiaozuo City (approval no. 20160518).

### Statistical Analysis

Statistical analyses were performed with SPSS 25.0 software (IBM Corp., Armonk, NY, USA). Continuous data were presented as means ± standard deviations. Measurement data between the two groups were conducted using independent samples *t*-test. Numeration data were analyzed using the *χ* ^2^ test. Variables with a non-normal distribution were expressed as median (P25 and P75) and were compared using the non-parametric Mann–Whitney *U* test. GraphPad Prism software was used to generate the receiver operating characteristic (ROC) curves of each index and its combined index in order to determine the sensitivity, specificity, optimal cutoff value, Youden index, negative predictive value (NPV), and the positive predictive value (PPV) of each index in patients infected with sepsis. The area under the ROC curve (AUC) was used to judge the accuracy of the test. The combined predictors of sIL-2R, TNF-α, and PCT were calculated with binary logistic regression analysis. The AUCs were compared between each index using *Z*-test. Spearman’s rank correlation coefficient was used to analyze the correlation between the levels of IL-2R, TNF-α, PCT and other laboratory parameters and APACHE II. Binary logistic regression analysis was applied to evaluate the risk predictive value of the levels of sIL-2R, TNF-α, and PCT for sepsis using median (P50) and quartiles (P25, P50, P75) as cut points, respectively. It was also used to calculate the values of the single-factor and multifactor adjusted odds ratios (AORs) and 95% confidence intervals (CI) based on maximum likelihood estimates. A *p* < 0.05 was considered statistically significant.

## Results

### Clinical Baseline of the Enrolled Subjects in Both Infection and Sepsis Groups

**Table 1** shows the clinical baseline of the 140 enrolled patients with closed abdominal injury complicated with severe multiple abdominal injuries. There was no statistical significance in the gender and age distribution between the infection group and the sepsis group (*p* > 0.05). The scores for WBC, CRP, SAA, IL-6, APACHE II, and SOFA in the sepsis group were all higher than those in the infection group, with statistical significance (*p* < 0.05).

**Table 1:** Characteristics of the enrolled patients.

Variables	Infection group (*n* = 70)	Sepsis group (*n* = 70)	*p-*value
Gender F/M)	38/32	41/29	0.609
Age (years)	72 (63,80)	78 (65,85)	0.099
Location of abdominal injury			0.994
Pancreas	6	7	
Duodenum	6	9	
Colon	13	19	
Liver	5	8	
Small intestine	9	10	
Spleen	20	28	
WBC (×10^9^/L)	12.22 (9.44–15.88)	17.50 (12.93–19.74)	<0.001
CRP (mg/L)	55.28 (25.23–91.31)	133.59 (85.20–225.50)	<0.001
SAA (mg/L)	76.54 (31.18–102.62)	124.61 (53.78–187.47)	0.001
IL-6 (pg/ml)	10.50 (4.34–29.93)	31.75 (17.93–113.20)	<0.001
sIL-2R	835.50 (658.25–1057.25)	1879.00 (1216.00–2872.50)	<0.001
TNF-α	11.45 (8.54–21.70)	31.20 (16.38–49.10)	<0.001
PCT	0.72 (0.47–1.87)	6.56 (1.75–8.62)	<0.001
SOFA score	0 (0–1)	10 (7–12)	<0.001
PaO_2_/FiO_2_ (mmHg)	452 (426–469)	287 (264–376)	<0.001
GCS	15 (15–15)	12 (11–14)	<0.001
MAP (mmHg)	90 (85–96)	66 (62–69)	<0.001
TBIL (μmol/L)	11.4 (9.2–15.3)	38.1 (27.8–68.6)	<0.001
PLT (×10^9^/L)	200 (176–232)	95 (83–126)	<0.001
CREA (μmol/L)	78.5 (67–97)	215 (134–319)	<0.001
APACHE II	10 (8–13)	18 (15–23)	<0.001

WBC, white blood cells; CRP, C-reactive protein; SAA, serum amyloid A; IL-6, interleukin 6; sIL-2R, soluble interleukin-2 receptor; TNF-α, tumor necrosis factor alpha; PCT, procalcitonin; SOFA, Sequential Organ Failure Assessment; GCS, Glasgow Coma Scale; MAP, mean arterial pressure; TBIL, total bilirubin; PLT, platelet; CREA, creatinine; APACHE II, Acute Physiology and Chronic Health Assessment.

### Expression Levels of sIL-2R, TNF-α, and PCT in the Two Groups of Subjects

**Figure 1:**
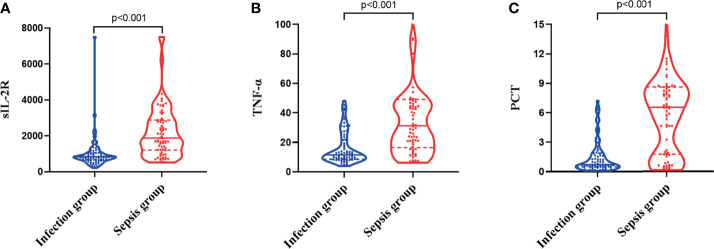
Differences in the expression levels of soluble interleukin-2 receptor (sIL-2R) **(A)**, tumor necrosis factor alpha (TNF-α) **(B)**, and procalcitonin (PCT) **(C)** between the infection group and the sepsis group.

The levels of sIL-2R in the sepsis group were higher than those in the infection group, and the difference was statistically significant (*Z* = −6.668, *p* < 0.001), as shown in **Figure 1A**. The levels of TNF-α in the sepsis group were higher than those in the infection group, and the difference was statistically significant (*Z* = −5.728, *p* < 0.001), as shown in **Figure 1B**. The levels of PCT in the sepsis group were higher than those in the infection group, and the difference was also statistically significant (*Z* = −6.560, *p* < 0.001), as shown in **Figure 1C**.

### Correlation Between sIL-2R, TNF-α, PCT, and Other Commonly Used Laboratory Infection Indicators in the Two Groups

**Figure 2:**
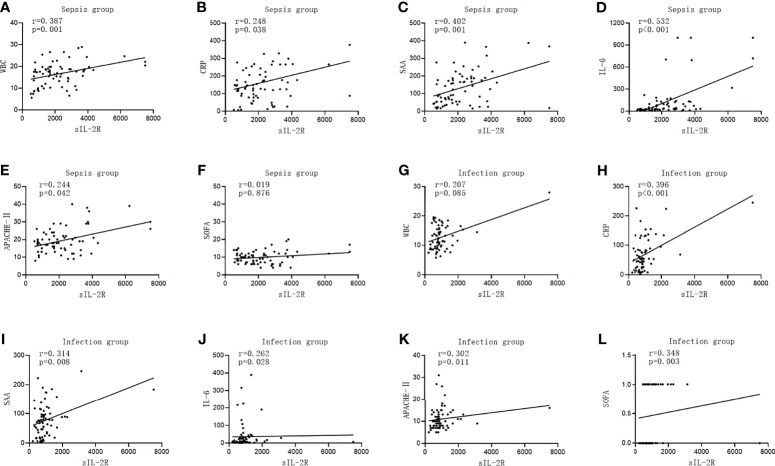
Correlations of soluble interleukin-2 receptor (sIL-2R) with the commonly used infection markers—white blood cells (WBC), C-reactive protein (CRP), serum amyloid A (SAA), interleukin 6 (IL-6), Acute Physiology and Chronic Health Assessment (APACHE II), and the Sequential Organ Failure Assessment (SOFA) score—in patients in the infection group and the sepsis group. **(A–F)** Correlations of sIL-2R with WBC **(A)**, CRP **(B)**, SAA **(C)**, IL-6 **(D)**, APACHE II **(E)**, and with the SOFA score **(F)** in patients in the sepsis group. **(G–L)** Correlations of sIL-2R with WBC **(G)**, CRP **(H)**, SAA **(I)**, IL-6 **(J)**, APACHE II **(K)**, and with the SOFA score **(L)** in patients in the infection group.

The level of sIL-2R was positively correlated with CRP, SAA, IL-6, APACHE II, and the SOFA score in the infection group (*r* = 0.396, *p* < 0.001; *r* = 0.314, *p* < 0.008; *r* = 0.262, *p* = 0.028; *r* = 0.302, *p* = 0.011; and *r* = 0.348, *p* = 0.003, respectively). There was no correlation between the level of sIL-2R and WBC (*r* = 0.207, *p* < 0.085). The levels of sIL-2R in the sepsis group were positively correlated with WBC, CRP, SAA, IL-6, and APACHE II (*r* = 0.387, *p* = 0.001; *r* = 0.248, *p* = 0.038; *r* = 0.402, *p* = 0.001; *r* = 0.532, *p* < 0.001; and *r* = 0.244, *p* = 0.042, respectively). No significant correlation between the level of sIL-2R and the SOFA score was observed for patients with sepsis (*r* = 0.019, *p* = 0.876, which is shown in **Figure 2**.

**Figure 3:**
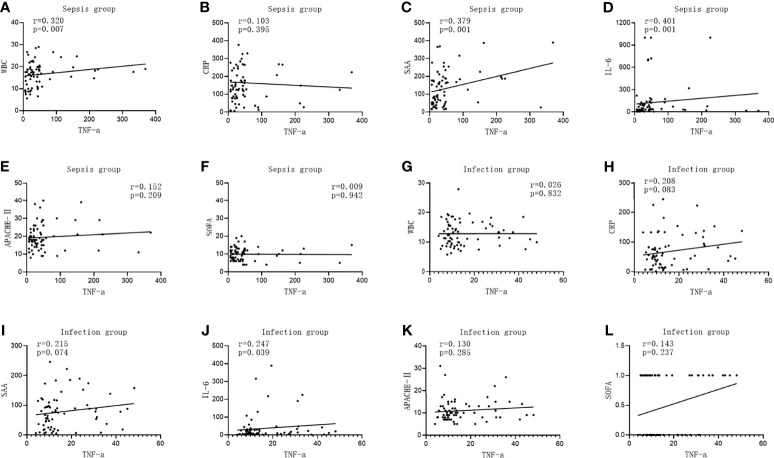
Correlations of tumor necrosis factor alpha (TNF-α) with the commonly used infection markers—white blood cells (WBC), C-reactive protein (CRP), serum amyloid A (SAA), interleukin 6 (IL-6), Acute Physiology and Chronic Health Assessment (APACHE II), and the Sequential Organ Failure Assessment (SOFA) score—in patients in the infection group and the sepsis group. **(A–G)** Correlations of TNF-α with WBC **(A)**, CRP **(B)**, SAA **(C)**, IL-6 **(D)**, APACHE II **(E)**, and with the SOFA score **(F)** in patients in the sepsis group. **(G–L)** Correlations of TNF-α with WBC **(G)**, CRP **(H)**, SAA **(I)**, IL-6 **(J)**, APACHE II **(K)**, and with the SOFA score **(K)** in patients in the infection group.

The TNF-α level in the infected group was positively correlated with IL-6 (*r* = 0.247, *p* = 0.039), while it was not correlated with WBC, CRP, SAA, APACHE II, and the SOFA score (*r* = 0.026, *p* = 0.832; *r* = 0.208, *p* = 0.083; *r* = 0.215, *p* = 0.074; *r* = 0.130, *p* = 0.285; and *r* = 0.143, *p* = 0.237, respectively). The TNF-α level was positively correlated with WBC, SAA, and IL-6 in the sepsis group (*r* = 0.320, *p* = 0.007; *r* = 0.379, *p* = 0.001; and *r* = 0.401, *p* = 0.001, respectively), but there was no correlation between the level of TNF-α and CRP, APACHE II, and the SOFA score (*r* = 0.103, *p* = 0.395; *r* = 0.152, *p* = 0.209; and *r* = 0.009, *p* = 0.942, respectively), as shown in **Figure 3**.

**Figure 4:**
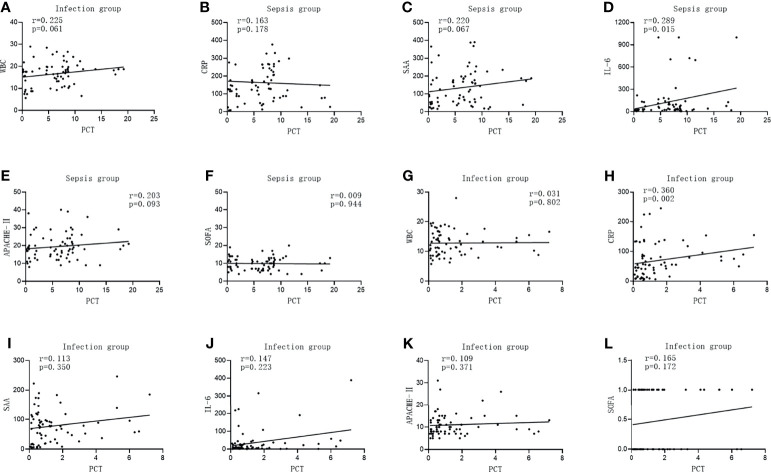
Correlations of procalcitonin (PCT) with the commonly used infection markers—white blood cells (WBC), C-reactive protein (CRP), serum amyloid A (SAA), interleukin 6 (IL-6), Acute Physiology and Chronic Health Assessment (APACHE II), and the Sequential Organ Failure Assessment (SOFA) score in patients in the infection group and sepsis group. **(A–G)** Correlations of PCT with WBC **(A)**, CRP **(B)**, SAA **(C)**, IL-6 **(D)**, APACHE II **(E)**, and with the SOFA score **(F)** in patients in the sepsis group. **(G–L)** Correlation of PCT with WBC **(G)**, CRP **(H)**, SAA **(I)**, IL-6 **(J)**, APACHE II, **(K)** and with the SOFA score **(L)** in patients in the infection group.

The PCT level in the infected group was positively correlated with CRP (*r* = 0.360, *p* = 0.002), while it was not correlated with WBC, SAA, IL-6, APACHE II, and the SOFA score (*r* = 0.031, *p* = 0.802; *r* = 0.113, *p* = 0.350; *r* = 0.147, *p* = 0.223; *r* = 0.109, *p* = 0.371; and *r* = 0.165, *p* = 0.172, respectively). The PCT level in the sepsis group was positively correlated with IL-6 (*r* = 0.289, *p* = 0.015), while it was not correlated with WBC, CRP, SAA, APACHE II, and the SOFA score (*r* = 0.225, *p* = 0.061; *r* = 0.163, *p* = 0.178; *r* = 0.220, *p* = 0.067; *r* = 0.203, *p* = 0.093; and *r* = 0.009, *p* = 0.944, respectively), which is shown in **Figure 4**.

### Diagnostic Performance of the Laboratory Infection Indicators in Subjects With Sepsis and Infection

Infection group Y (sepsis group = 1, infection group = 0) was used as the dependent variable; sIL-2R(X1), TNF-α(X2), and PCT(X3) were used as the independent variables. The joint predictors of sIL-2R, TNF-α, and PCT were calculated using binary logistic regression analysis, and the regression equation was *Y* = −2.343 + 0.000X1 + 0.026X2 + 0.364X3. The joint predictors were used as three joint test indexes to analyze the results.

**Figure 5:**
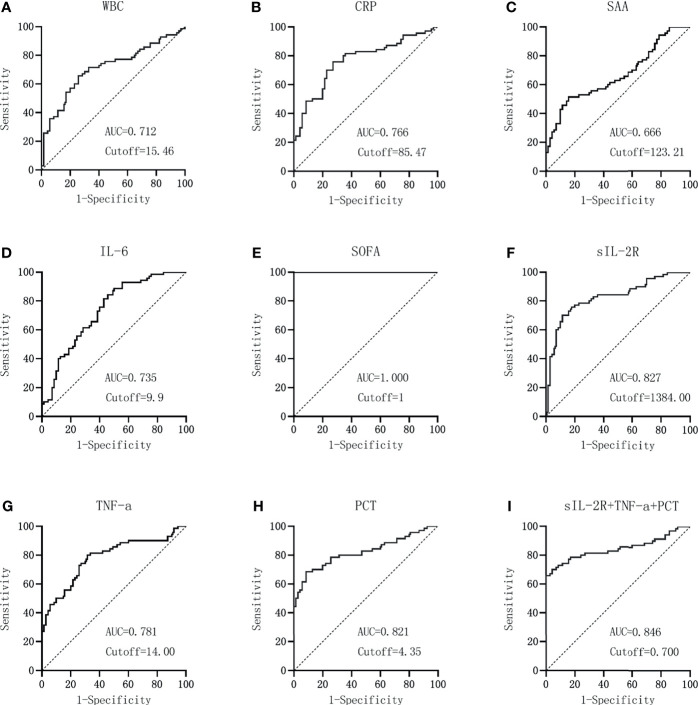
Diagnostic value of the laboratory infection markers in patients in the sepsis group *versus* the infection group. **(A–I)** Diagnostic value of white blood cells (WBC) **(A)**, C-reactive protein (CRP) **(B)**, serum amyloid A (SAA) **(C)**, interleukin 6 (IL-6) **(D)**, Sequential Organ Failure Assessment (SOFA) score **(E)**, soluble interleukin-2 receptor (sIL-2R) **(F)**, tumor necrosis factor alpha (TNF-α) **(G)**, procalcitonin (PCT) **(H)**, and the combined sIL-2R+TNF-a+PCT **(I)** in patients in the sepsis group *versus* the infection group.

GraphPad Prism was used to plot the ROC curves of each index and the combined test, as shown in **Figures 5A–I**. When the AUC of WBC is 0.712 and the cutoff value is 15.46 × 10^9^/L, the sensitivity and specificity were 65.71% and 74.29% and the NPV and PPV were 68.4% and 71.9%, respectively. For CRP detection, when the AUC is 0.766 and the cutoff value is 85.47 mg/L, the sensitivity and specificity were 75.71% and 72.86% and NPV and PPV are 75.0% and 73.6%, respectively. When the AUC is 0.666 and the cutoff value is 123.21 mg/L, the sensitivity and specificity were 51.43% and 84.29% and the NPV and PPV are 63.4% and 76.6%, respectively. IL-6 detection with an AUC of 0.735 and a cutoff value of 9.9 pg/ml showed sensitivity and specificity values of 88.57% and 50.00% and NPV and PPV of 81.4% and 63.9%, respectively. When the AUC of the SOFA score is 1.000 and the cutoff value is 1, the sensitivity and specificity were 100% and 100% and the NPV and PPV were 100% and 100%, respectively. For sIL-2R detection, an AUC of 0.827 and a cutoff value of 1384 U/ml produced sensitivity and specificity values of 70.00% and 88.57% and NPV and PPV of 74.7% and 86.0%, respectively. When the AUC of TNF-α detection is 0.781 and the cutoff value is 14.00 pg/ml, the sensitivity and specificity were 80.00% and 68.57% and the NPV and PPV were 77.4% and 71.8%, respectively. For PCT, when the AUC is 0.821 and the cutoff value is 4.35 ng/ml, the sensitivity and specificity were 68.57% and 91.43% and the NPV and PPV were 74.4% and 88.9%, respectively. The sensitivity and specificity were 70.00% and 95.71% and the NPV and PPV were 76.1% and 94.2%, respectively, for sIL-2R+TNF-α+PCT when the AUC is 0.846 and the cutoff value is 0.70, which are shown in **Table 2**.

**Table 2:** Diagnostic performance of the laboratory infection indicators in subjects with sepsis and infection.

Variables	Youden index	Cutoff	AUC	Sensitivity (%)	Specificity (%)	AUC 95%CI	NPV (%)	PPV (%)
WBC	0.400	15.46	0.712	65.71	74.29	0.629–0.785	68.4	71.9
CRP	0.486	85.47	0.766	75.71	72.86	0.687–0.833	75.0	73.6
SAA	0.357	123.21	0.666	51.43	84.29	0.581–0.743	63.4	76.6
IL-6	0.386	9.90	0.735	88.57	50.00	0.654–0.806	81.4	63.9
SOFA	1.000	1.00	1.000	100	100	0.974–1.000	100.0	100.0
sIL-2R	0.586	1,384.00	0.827	70.00	88.57	0.753–0.885	74.7	86.0
TNF-α	0.486	14.00	0.781	80.00	68.57	0.703–0.846	77.4	71.8
PCT	0.600	4.35	0.821	68.57	91.43	0.748–0.881	74.4	88.9
sIL-2R+TNF-α+PCT	0.657	0.70	0.846	70.00	95.71	0.775–0.901	76.1	94.2

WBC, white blood cells; CRP, C-reactive protein; SAA, serum amyloid A; IL-6, interleukin 6; SOFA, Sequential Organ Failure Assessment; sIL-2R, soluble interleukin-2 receptor; TNF-α, tumor necrosis factor alpha; PCT, procalcitonin; AUC, area under the ROC curve; NPV, negative predictive value; PPV, positive predictive value.

According to the data in **Table 2**, the combination of sIL-2R, TNF-α, PCT, and sIL-2R+TNF-a+PCT had higher AUCs and better diagnostic performance. MedCalc software was used to compare the AUCs of sIL-2R, TNF-a, PCT, and sIL-2R+TNF-α+PCT. The AUCs of sIL-2R and TNF-α, sIL-2R and PCT, and TNF-α and PCT were also compared. There was no statistically significant difference in the AUCs between sIL-2R and PCT (*p* > 0.05). The AUC of the combined test was greater than that of TNF-α, and the difference was statistically significant (*p* < 0.05), as shown in **Table 3**.

**Table 3:** Comparison of AUC areas for sIL-2R, TNF-α, PCT and sIL-2R+TNF-α+PCT combined assays.

Variables	*Z*-value	*p*-value
Three combined assays compared with sIL-2R	0.851	0.395
Three combined assays compared with TNF-α	2.355	0.019
Three combined assays compared with PCT	1.160	0.246
sIL-2R and TNF-α	1.386	0.166
sIL-2R and PCT	0.162	0.871
TNF-α and PCT	1.021	0.307

AUC, area under the ROC curve; sIL-2R, soluble interleukin-2 receptor; TNF-α, tumor necrosis factor alpha; PCT, procalcitonin.

### Risk Assessment of sIL-2R, TNF-α, and PCT in Predicting Sepsis in Patients With Closed Abdominal Injury Complicated With Severe Multiple Abdominal Injuries

**Figure 6:**
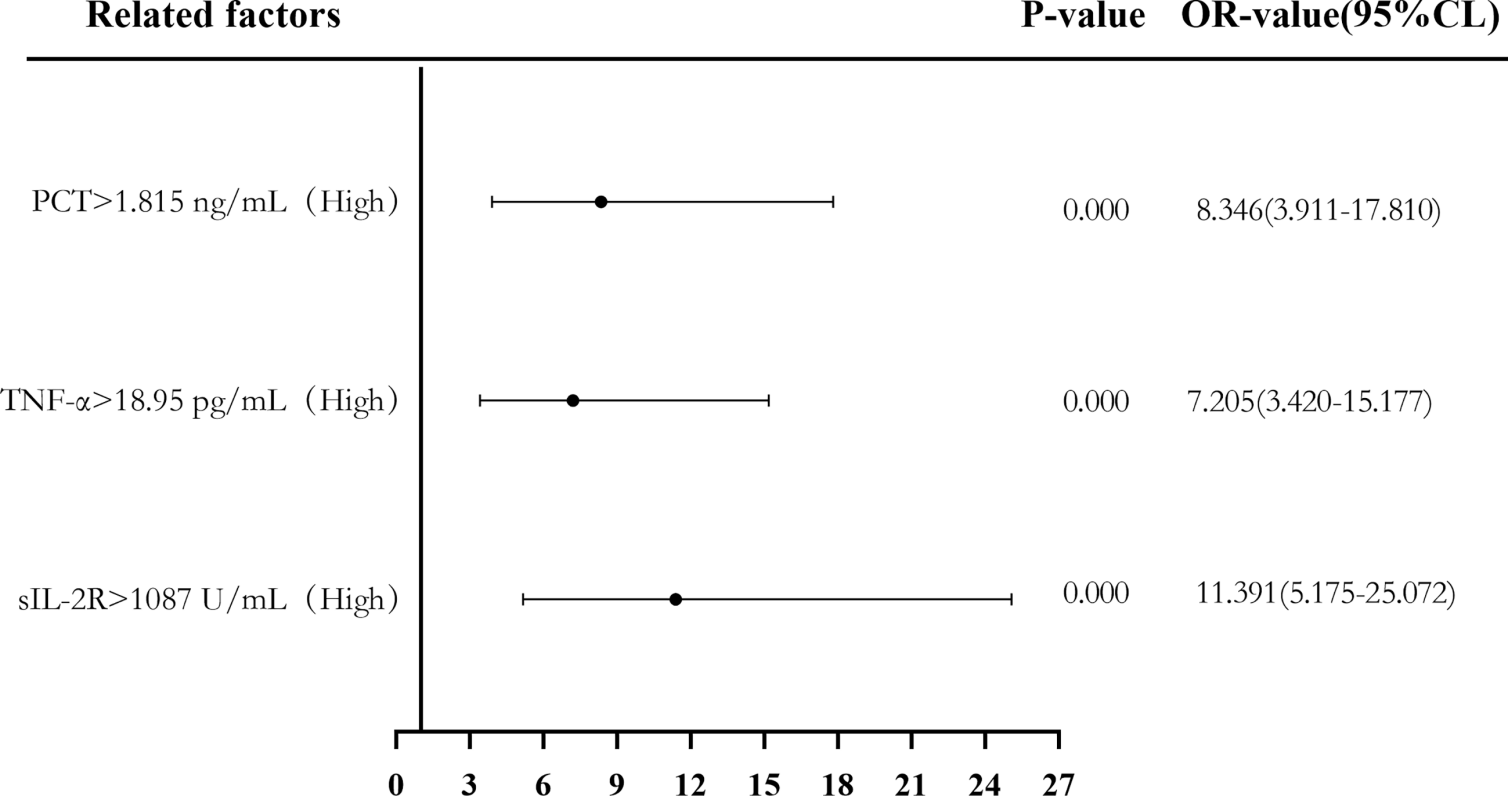
Forest plot of the univariate logistic regression analysis of soluble interleukin-2 receptor (sIL-2R), tumor necrosis factor alpha (TNF-α), procalcitonin (PCT), and infection in patients with sepsis.

**Figure 7:**
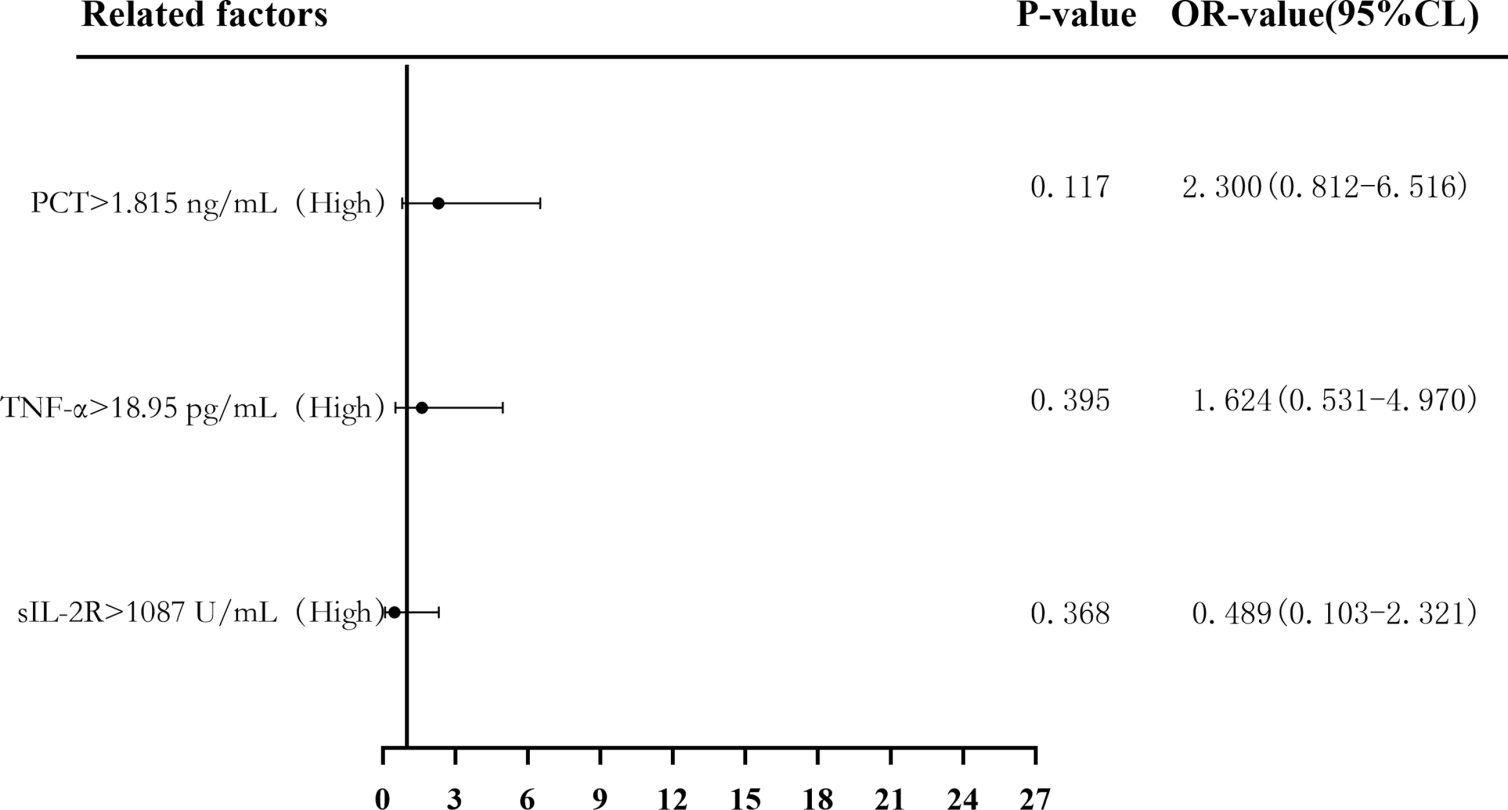
Forest plot of the multifactorial logistic regression analysis of soluble interleukin-2 receptor (sIL-2R), tumor necrosis factor alpha (TNF-α), procalcitonin (PCT) and infection in patients with sepsis. Multifactorial correction included the following variables: sIL-2R, TNF-α, PCT, white blood cells (WBC), C-reactive protein (CRP), serum amyloid A (SAA), interleukin 6 (IL-6), and Acute Physiology and Chronic Health Assessment (APACHE II).

Binary logistic regression analysis was used to evaluate the risk predictive value of the levels of sIL-2R, TNF-α, and PCT in the sepsis group. The median cutoff point (two classifications) and the quartiles (P25, P50, and P75) were evaluated as cutoff points (four classifications). Firstly, patients were divided into a low-level group and a high-level group according to the median values of sIL-2R (1,087 U/ml), TNF-α (18.95 pg/ml), and PCT (1.815 ng/ml). Compared with low sIL-2R, the risk of sepsis in patients with high sIL-2R was 11.391 (95%CI = 5.175–25.072, *p* < 0.05), and the adjusted OR was 0.489 (95%CI = 0.103–2.321, *p* > 0.05). Compared with low TNF-α, the risk of sepsis in patients with high TNF-α levels was 7.205 (95%CI = 3.420–15.177, *p* < 0.05), and the adjusted OR was 1.624 (95%CI = 0.531–4.970, *p* > 0.05). Compared with low PCT, the risk of sepsis in patients with high PCT was 8.346 (95%CI = 3.911–17.810, *p* < 0.05), and the adjusted OR was 2.300 (95%CI = 0.812–6.516, *p* > 0.05), which can be seen in **Figures 6** and **7**.

**Figure 8:**
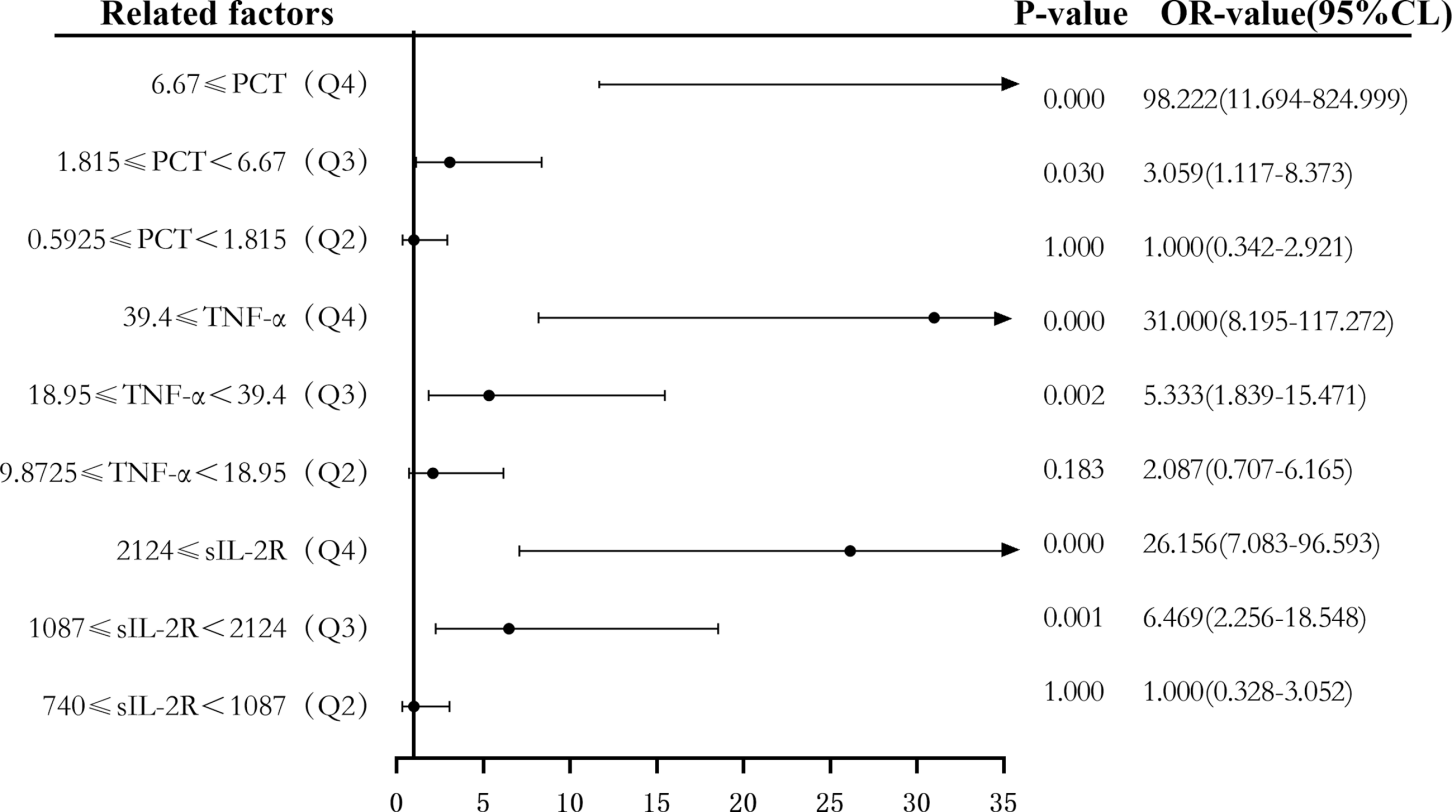
Forest plot of the univariate logistic regression analysis of soluble interleukin-2 receptor (sIL-2R), tumor necrosis factor alpha (TNF-α), procalcitonin (PCT) and infection in patients with sepsis.

**Figure 9:**
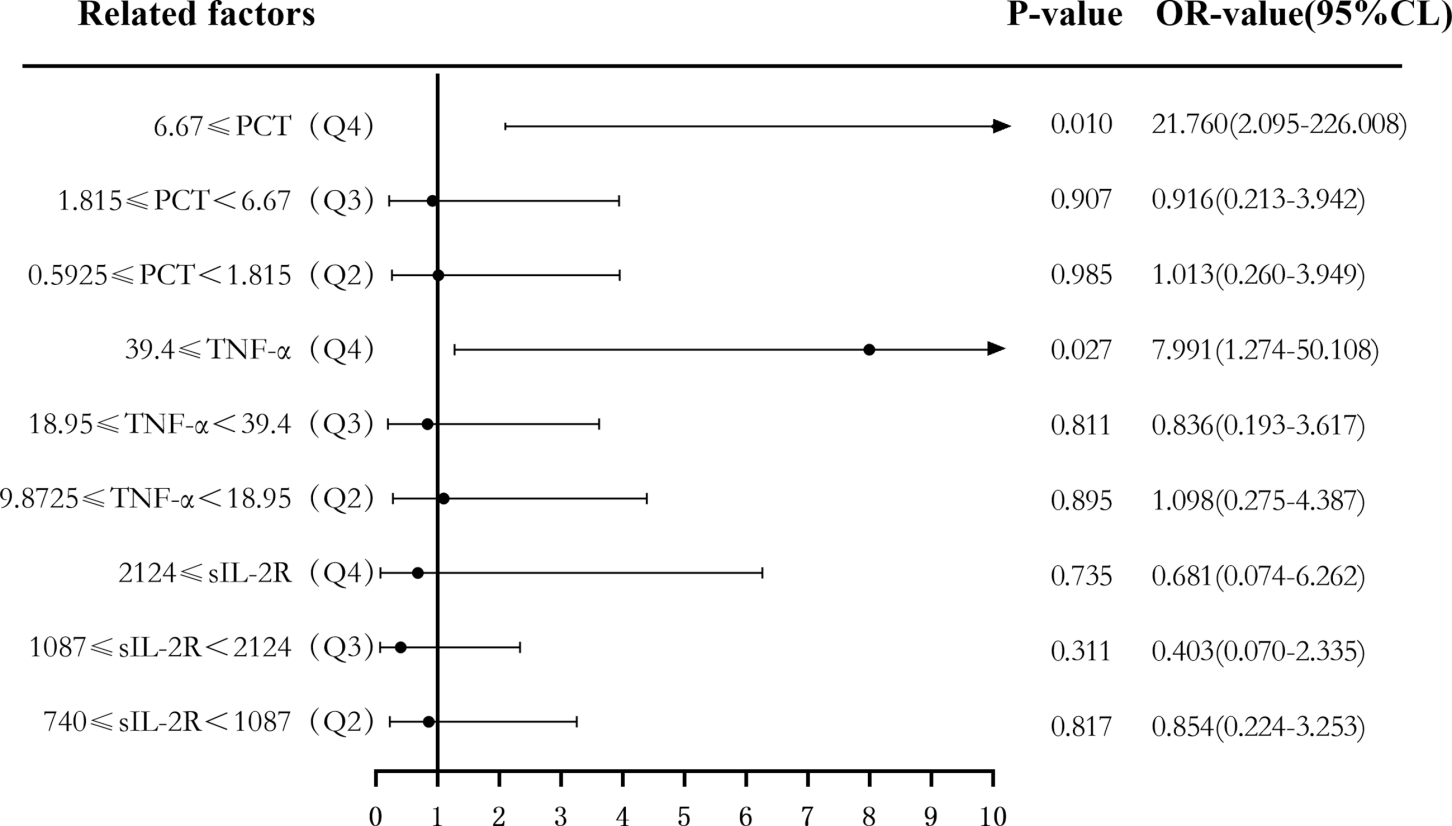
Forest plot of the multifactor logistic regression analysis of soluble interleukin-2 receptor (sIL-2R), tumor necrosis factor alpha (TNF-α), procalcitonin (PCT), and infection in patients with sepsis. Multifactor-corrected variables included: sIL-2R, TNF-α, PCT, white blood cells (WBC), C-reactive protein (CRP), serum amyloid A (SAA), interleukin 6 (IL-6), Acute Physiology and Chronic Health Assessment (APACHE II).

Secondly, according to the quartile values of sIL-2R (Q1 < 740, 740 ≤ Q2 < 1,087, 1,087 ≤ Q3 < 2,124, and 2,124 ≤ Q4), TNF-α (Q1 < 9.8725, 9.8725 ≤ Q2 < 18.95, 18.95 ≤ Q3 < 39.4, and 39.4 ≤ Q4), PCT (Q1 < 0.5925, 0.5925 ≤ Q2 < 1.815, 1.815 ≤ Q3 < 6.67, and 6.67 ≤ Q4), the patients were divided into the Q1, Q2, Q3, and Q4 groups from low to high levels. Compared to the group with the lowest sIL-2R levels (Q1), the ORs of sepsis risk in the Q2, Q3, and Q4 groups were 1.000 (0.328–3.052), 6.469 (2.256–18.548), and 26.156 (7.083–96.593), respectively. The ORs after correction were 0.854 (0.224–3.253), 0.403 (0.070–2.335), and 0.681 (0.074–6.262), respectively. Compared to the group with the lowest TNF-α levels (Q1), the ORs of sepsis risk in the Q2, Q3, and Q4 groups were 2.087 (0.707–6.165), 5.333 (1.839–15.471), and 31.000 (8.195–117.272), respectively. The ORs after correction were 1.098 (0.275–4.387), 0.836 (0.193–3.617), and 7.991 (1.274–50.108), respectively. Compared to the group with the lowest PCT levels (Q1), the ORs of sepsis risk in the Q2, Q3, and Q4 groups were 1.000 (0.342–2.921), 3.059 (1.117–8.373), and 98.222 (11.694–824.999), respectively. The corrected ORs were 1.013 (0.260–3.949), 0.916 (0.213–3.942), and 21.760 (2.095–226.008), as shown in **Figures 8** and **9**.

## Discussion

As an important complication of closed abdominal injury and severe multiple abdominal injuries, sepsis is a complex disease caused by the body’s dysfunctional response to infection and is associated with acute organ dysfunction and a high risk of death (19, 20). Sepsis can lead to a global public health emergency, affecting millions of people worldwide and being one of the largest causes of death in the world (21). What plays an essential role in the treatment of sepsis is the early removal of infected lesions and the use of antibiotics as quickly and accurately as possible (22). Mortality is significantly increased for each hour of delay in antibiotic administration (23, 24), and the delay in antibiotic administration was associated with prolonged length of hospital stay, severity of organ dysfunction, and adverse clinical outcomes (25). However, for all patients with suspected sepsis, antibiotics given within 1 h will lead to its unreasonable use and to increased bacterial resistance (26–28). Therefore, early identification and diagnosis of sepsis patients have become particularly important (29).

In this study, we analyzed the diagnostic value of sIL-2R, TNF-α, and PCT in sepsis patients. The results showed that sIL-2R, TNF-α, and PCT in the sepsis group were significantly higher than those in the infection group, which was consistent with previous studies (8–10, 12, 13) on infectious diseases. It also showed a certain diagnostic value in the degree of infection in patients with closed abdominal injury combined with severe multiple injuries.

The level of sIL-2R in sepsis patients was positively correlated with WBC, CRP, SAA, IL-6, and APACHE II, and it was also positively correlated with CRP, SAA, IL-6, APACHE II, and the SOFA score in the infection group, indicating that the level of sIL-2R could better reflect the indicators related to inflammation. Similarly, studies have shown that interleukin is an important cytokine released by immune cells *in vivo*, which can regulate immune response and call immune cells to the site of infection, and interleukin presents an inflammatory response after activation of the complement pathway (30). The level of TNF-a was positively correlated with WBC, SAA, and IL-6 in the sepsis group and was also positively correlated with IL-6 in the infection group, which also indicated that the level of TNF-α could better reflect the progression of inflammation; the correlation between the TNF-α level of the sepsis group was better than that of the infection group. TNF-α plays a central role in systemic inflammatory response due to its ability to release other cytokines in the early stage of infection and its direct influence on septic shock, and the plasma levels of TNF-α are associated with sepsis-induced death (31). The level of PCT was positively correlated with IL-6 only in the sepsis group and CRP only in the infection group. The results showed that the levels of sIL-2R, TNF-α, and PCT were correlated with other laboratory indicators of infection in the two groups of patients. Only sIL-2R and TNF-α in patients with sepsis related to other laboratory indicators were higher than PCT, which may be related to differences in the sensitivity and specificity between projects.

The ROC curve of each index and the combined test generated by GraphPad Prism software showed that the AUCs of sIL-2R, TNF-α, PCT, and sIL-2R+TNF-α+PCT were higher than those of WBC, CRP, SAA, and IL-6, which is basically consistent with the study by Spiegel et al. (28), indicating that the sIL-2R, TNF-α, and PCT indexes are better than the routine laboratory infection monitoring indexes in terms of diagnostic value in infectious diseases, despite lower than the SOFA score. Comparison of the AUCs of sIL-2R, TNF-α, PCT, and sIL-2R+TNF-α+PCT showed that there was no difference between the AUCs of the three combined tests and those of sIL-2R and PCT, indicating that the three combined tests were not better than sIL-2R and PCT alone in terms of diagnostic performance. PCT still had good sensitivity and specificity in the diagnosis of sepsis caused by closed abdominal injury combined with severe multiple abdominal injury, and sIL-2R detection also had good diagnostic ability. It should be noted that the PPV of the three joint tests reached 94.2%, so these joint tests can be carried out to increase the positive predictive ability, if conditions permit.

The risk predictive value of the levels of sIL-2R, TNF-α, and PCT for sepsis was assessed using binary logistic regression analysis, with median cutoff points (two classifications) and quartiles (P25, P50, and P75) as cutoff points (four classifications). The results showed that the corrected sIL-2R, TNF-α, and PCT in the high-level group was not superior to those in the low-level group when the median cut point was used for the classification of the two groups. When the four groups were classified using quantiles as cut points, the OR risk values of the high levels of TNF-α and PCT (Q4) and the low level of PCT (Q1) after correction were 7.991 and 21.76, respectively, the difference being statistically significant. There was no significant difference between the other groups and the low-level group (Q1). The results showed that when PCT ≥ 6.67 and TNF-α ≥ 39.4, they could be used as predictors of the risk of sepsis.

## Conclusions

The detection of sIL-2R, TNF-α, and PCT in patients with sepsis has good value for the diagnosis of sepsis infection in patients with closed abdominal injury complicated with severe multiple abdominal injuries. High concentrations of PCT and TNF-α can be used as predictors of the risk of septic infection.

## Data Availability Statement

The original contributions presented in the study are included in the article/supplementary material. Further inquiries can be directed to the corresponding authors.

## Ethics Statement

The studies involving human participants were reviewed and approved by the ethics committees of the Fifth People’s Hospital of Jiaozuo City (approval no. 20160518). Written informed consent to participate in this study was provided by the participants’ legal guardian/next of kin.

## Author Contributions

JW, WZ, and ZX contributed to the study concept and design, acquisition of data, analysis and interpretation of data, and drafting of the manuscript. W-wW and G-hZ contributed to statistical analysis. L-zH contributed to sample collections. A-qS and G-hZ contributed to the study concept and design, study supervision, and critical revision of the manuscript. All authors contributed to the article and approved the submitted version.

## Funding

This work was supported by the National Natural Science Foundation of China (no. 81802084) and Jiaozuo science and technology project (no. 2020162).

## Conflict of Interest

The authors declare that the research was conducted in the absence of any commercial or financial relationships that could be construed as a potential conflict of interest.

## Publisher’s Note

All claims expressed in this article are solely those of the authors and do not necessarily represent those of their affiliated organizations, or those of the publisher, the editors and the reviewers. Any product that may be evaluated in this article, or claim that may be made by its manufacturer, is not guaranteed or endorsed by the publisher.

## Abbreviations

sIL-2R, soluble interleukin-2 receptor; TNF-α, tumor necrosis factor-α; PCT, procalcitonin; APACHE II, Acute Physiology and Chronic Health Assessment; NPV, negative predictive value; PPV, positive predictive value; AUC, area under the curve; AOR, adjusted odds ratios; CI, confidence intervals.
